# Selection signatures and formation of the Samosir goat breed through the cultures of the Batak Toba Tribe in Samosir Island, Indonesia

**DOI:** 10.14202/vetworld.2022.1044-1050

**Published:** 2022-04-24

**Authors:** Suhendra Pakpahan, Rini Widayanti, Wayan T. Artama

**Affiliations:** 1 Museum Zoologicum Bogoriense, Research Center for Applied Zoology, National Research and Innovation Agency (BRIN), Cibinong, West Java, Indonesia; 2 Department of Biochemistry and Molecular Biology, Faculty of Veterinary Medicine, Universitas Gadjah Mada, Yogyakarta, Indonesia

**Keywords:** Batak tribe, ethnobiology, mitochondrial DNA, phylogenetic analysis, Samosir goat

## Abstract

**Background and Aim:**

The Samosir goat has a high cultural value and is a source of germplasm in Indonesia. This study aimed to reveal the history and selection signatures of the Samosir goat.

**Materials and Methods:**

A total of 25 goats were divided into seven subpopulations of Indonesian goat breeds. Deoxyribonucleic acid (DNA) from blood samples was isolated with the use of the gSYNC™ DNA Mini Kit (Geneaid, Taipei, Taiwan). Cytb gene amplification was performed by the polymerase chain reaction (PCR) method, and the PCR products were sequenced. A phylogenetic tree was constructed by the neighbor-joining method using MEGA 11 software. A questionnaire was used to collect information related to the history and breeding practices of the Samosir goat on Samosir Island.

**Results:**

Samosir goats are divided into four groups based on their coat color: Completely white, white with brown spots, white with black spots, and white with brown and black spots. The body form of the Samosir goat is similar to that of the Kacang goat. The space below a traditional Toba Batak house is used as a goat pen. The genetic difference between the Samosir goat and the Kacang goat based on the Cytb gene was approximately 0.1%.

**Conclusion:**

Phylogenetic analysis between Samosir goats and other indigenous Indonesian goats revealed that Samosir goats form a single clade, with a very close genetic distance from other local goats, such as the Kacang goat. The Toba Batak culture on Samosir Island has significantly influenced the selection and formation of the Samosir goat breed.

## Introduction

The Samosir goat contains a wealth of germplasm in Indonesia that needs to be developed and preserved. This breed is found only on Samosir Island and has a distinct phenotype known locally as the white goat or Batak goat [1,2]. The Batak tribal people who live on Samosir Island continue to keep this goat because it serves several purposes as a source of income, a source of animal protein, and use in Batak tribal cultural events [3]. The Samosir goat is believed to be used in traditional medicine as well [4]. The Samosir goat has been kept by local residents for generations on Samosir Island in the middle of Lake Toba, Samosir Regency, North Sumatra Province [1].

The preservation of genetic variation and evolutionary potential to ensure the long-term survival of a species is a key paradigm in conservation biology [5]. This is especially important in small and dispersed populations with low genetic diversity that is at risk of extinction due to inbreeding depression and loss of genetic diversity [6]. The Samosir goat shares almost all of the characteristics of other local Indonesian goats (body weight and length), except that the prevailing coat color in the Samosir goat population is white [7]. The Samosir goat has a very close genetic distance to the Kacang goat, a native local Indonesian goat that has the longest history of adaptation [7,8]. The Samosir goat was designated as a wealth of local Indonesian livestock genetic resources by the Indonesian Minister of Agriculture through the Decree of the Minister of Agriculture Number 40 of 2017.

Goats are the result of the domestication of wild animals [9]. Domestication of goats is thought to have occurred in mountainous areas of West Asia during the 9^th^ to the 7^th^ centuries BC [10]. Initially, goats were domesticated for meat [11]. Goats are also considered the oldest dairy animals due to the ease with which they are milked [12]. Some local goat germplasm in Indonesia are the Marica, Samosir, Muara, Kosta, Gembrong, Peranakan Etawah, Kacang, and Benggala goat [7,13]. At present, the population of Samosir goats has declined due to various factors, including local culture [14].

The Batak Toba is a North Sumatra tribe that lives on the island of Samosir and its surroundings [15]. In ancient times, the Batak tribe practiced an animistic religion known as Parmalim, which lasted so long in the Batak tribe that many relics became habits and persisted in the original Batak culture [16,17]. The goats used for ceremonial events are males with white bodies, heads, legs, horns, and hooves [1]. The Parmalim religion has existed on Samosir Island since the beginning of the Batak tribe civilization. There are many relics in the form of notes and direct interviews with the community indicating that the Batak tribe from Samosir Island is known as *Sianjur Mulamula* [18,19].

Goats are one of the favorite types of livestock for the people of Samosir, in addition to buffalo, cows, and pigs. Goats have an important role for the people of Samosir, especially in the celebration of traditional local culture and religion. There have been no studies of the influence of Batak culture and customs on the breeding of the Samosir goat. Therefore, this study was performed to reveal the history and selection signatures of the Samosir goat.

## Materials and Methods

### Ethical approval and Informed consent

This study was approved by the Animal Ethics Committee for using Animal and Scientific Procedures in the Faculty of Veterinary Medicine, Universitas Gadjah Mada, Indonesia (No. 003/EC-FKH/Eks). Verbal informed consent was obtained from each participant.

### Study period and area

**Figure-1:**
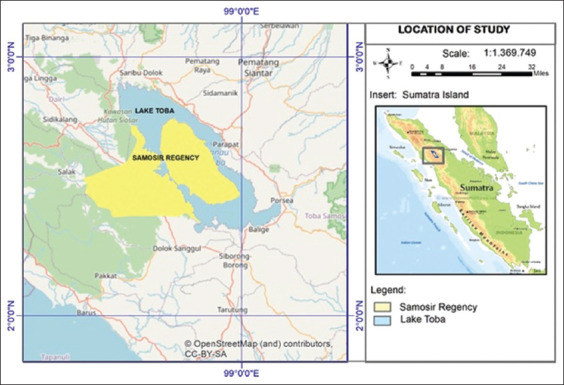
Map of Samosir Regency and Samosir goat distribution. The OpenStreetMap provides free geographic data and is available under the Open Database License [https://www.openstreetmap.org/copyright].

The study was conducted from January to November 2021. Samosir Regency is geographically located at latitude 2° 24’-2° 25’ North and longitude 98° 21’-99° 55‘ East. The territory of Samosir Regency is administratively flanked by seven regencies, namely, in the north, it borders Karo Regency and Simalungun Regency; in the east, it is bordered by Toba Samosir Regency; in the south, it is bordered by North Tapanuli Regency and Humbang Hasundutan Regency; and in the west, it is bordered by Dairi Regency and West Pakpak Regency. The area of Samosir Regency is 2069.05 km^2^, the land area is 1444.25 km^2^, and the lake area is 624.80 km^2^ (Figure-1). Characteristics of surface and slope Samosir Regency is located in a highland area, with an altitude between 700 and 1700 m above sea level, with the composition; 700-1000 m asl=±10 %; 1000-1500 m asl=±25%; and >1500 m asl=±65%. With the slope composition as follows: 0-20 km^2^ (flat)=±10%; 2-150 km^2^ (sloping)=±20%; 15-400 km^2^ (oblique)=±55%; >400 km^2^ (steep)=±15%. Types of topographic soil and soil contours in Samosir Regency are generally hilly and undulating.

### Sample collection

The coat color of Samosir goat was the main subject of this research. However, to determine the genetic distance and relationship with local Indonesian goats of the Samosir goat, several other types of Indonesian local goats were included in the study. Blood samples (3 mL) were taken from each type of goat to obtain deoxyribonucleic acid (DNA) material. The blood samples were collected from 25 goats divided into seven subpopulations of Indonesian goat breeds: Samosir goat from Samosir Regency (n=5), Muara goat from North Tapanuli Regency (n=3), Peranakan Etawah goat from Yogyakarta province (n=3), Jawarandu goat from Yogyakarta province (n=3), Kacang goat from East Java (n=5), Gembrong goat from Bali province (n=3), and Marica goat from South Sulawesi (n=3).

### Ethnobiological information

A questionnaire was used to collect information about the history and breeding practices of the Samosir goat on Samosir Island. We visited and observed the Samosir goat population on Samosir Island. Information on the population and distribution of Samosir goats was obtained from the Department of Agriculture and Livestock of Samosir Regency. Data and information about Samosir goat domestication were obtained from interviews with community members. Ethnozoological information about the Samosir goat was collected from 15 interviewees in Pangururan, Palipi, Ronggur Nihuta, and Onan Runggu districts. Traditional leaders and elders were chosen as sources of information about the history of the maintenance and function of the Samosir goat. The interviews were conducted in the Batak Toba language.

### Molecular techniques

The blood samples were isolated with the use of the gSYNC™ DNA Mini Kit (Geneaid Biotech Ltd., Taipei, Taiwan). The primers of goat mtDNA Cytb were as follows: SCF: 5’-GGAATCTAACCATGACCAAT-3’ and SCR: 5’-GCTTCTTCCTTGAGTCTTAG-3’ [8]. Cytb gene amplification was performed by the polymerase chain reaction (PCR) method, and the PCR products were visualized by electrophoresis using agarose gel at a concentration of 1%.

### Phylogenetic analysis

MEGA 11 version 10.2 was used to analyze the Cytb gene sequences. Indonesian goat Cytb gene sequences were aligned with *Capra aegagrus* (ID: AB004069.1) Cytb sequences from the National Center for Biotechnology Information database using ClustalW in the MEGA program. A phylogenetic tree was constructed by the neighbor-joining method. The percentage of replicate trees in which the associated taxa clustered together in the bootstrap test (1000 replicates) was calculated. Evolutionary analyses were performed in MEGA 11 [20]. Evolutionary distances were computed by the p-distance method [21].

## Results

### Samosir goat phenotype

**Figure-2:**
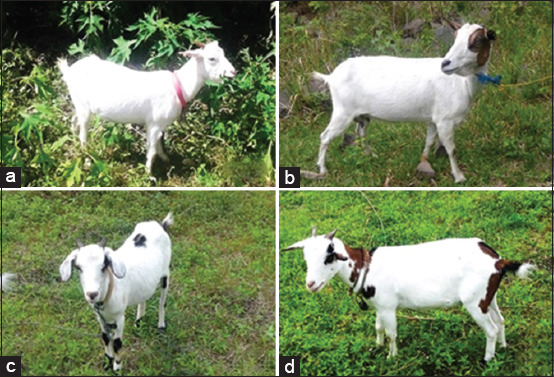
Group of Samosir goats based on coat colors, white (a), white-brown (b), white-black (c), and white-brown-black (d).

The Samosir goat has a body form similar to that of the Kacang goat, a local goat scattered throughout the Indonesian archipelago. The predominant coat color of the Samosir goat is white, and this characteristic differentiates the Samosir goat from other Indonesian local goats. The coat color of some Samosir goats is a mixture of white, brown, and black. Samosir goats are divided into four groups based on their coat color: Completely white, white with brown spots, white with black spots, and white with brown and black spots (Figure-2). The area of colors other than white varies greatly, but the color is always dominated by white.

### History of Samosir goat breeding

**Figure-3:**
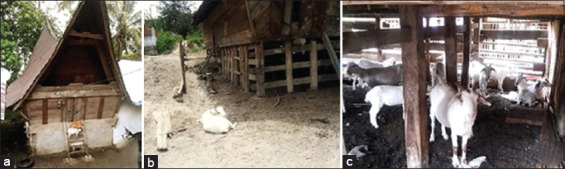
The Batak Toba house and Samosir goat breeding, custom house (a), home yard (b), and underneath the house as a goat pen.

The traditional Batak Toba house (*Rumah Bolon*) is built on stilts and is made of wood and rattan. The stilt house structure is used for protection against wild animals, and the pit is used as a cage for goats and other livestock, such as buffalo, cows, and pigs (Figure-3). During the day, goats are taken into the fields or forests and released to graze. At night, the goats are brought back to the area under the house. The Toba Batak people’s main sources of income are farming, livestock raising, and fishing in the lake.

The Samosir goat is an endemic goat that is found only in the Samosir Regency. At the beginning of civilization on the island of Samosir, this animal was used for the agreement of the king of Batak (*Parpadanan*). The king was presented with a male goat that was white all over his body, including the hair, hooves, horns, and eyes. The male white goat was called *Hambing Puti* and the female was called*Hambing Bontar*. In ceremonial events, only male white goats were offered.

**Figure-4:**
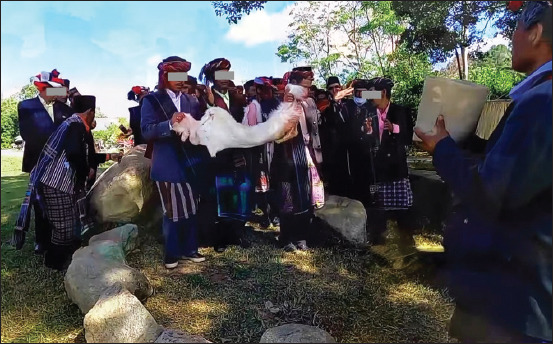
The ritual of offering white goats to *Mula Jadi Na Bolon* (God Almighty).

In the past, the Batak Toba people believed that a clean white pet symbolized purity or innocence. White pets also included native chickens, pigs, and others. Before the spread of Christianity and Islam to the Batak area, many Batak people performed worship ceremonies to ask for blessings and health. The Batak community began at Pusuk Buhit, Samosir Regency, and therefore, worship ceremonies were performed on Mount Pusuk Buhit (Figure-4). This ceremony is still performed, especially by people who adhere to a belief called *Parmalim*, although with the spread of religion to Batak Land, its performance is decreasing. At this time, the Batak people still believe that Mount Pusuk Buhit has mystical powers, so in this area, it is not allowed to say or do indecent things.

Samosir goats can also be found outside the island of Samosir, because they are traded in markets outside Samosir, such as North Tapanuli, Humbang Hasundutan, and Karo regencies. If people want to hold a worship ceremony, they will look for a white goat everywhere. The white goat is believed to be an incarnation of the Almighty. It is usually born to a low-income family. The goat brings extraordinary good fortune to the family that owns it. It is significantly more valuable than ordinary goats. Its price can reach millions, depending on the agreement of the buyer and seller. The family’s crops and livestock will thrive and their income will be abundant. This goat cannot be produced intentionally by people in the community. Many people keep goats in large numbers but never see the birth of a white goat.

### Parmalim as a first Batak Toba religion

**Figure-5:**
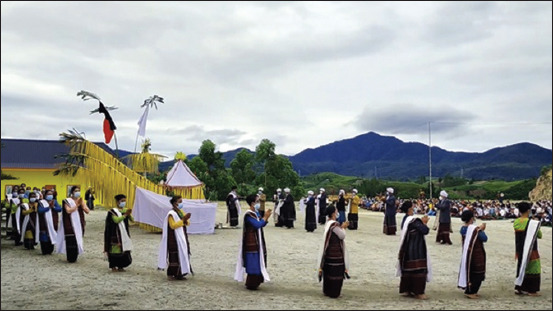
One of the Parmalim religious rites (*Sipaha Lima*).

Parmalim is a religion that was born in Batak Land and still exists as a role model for some of the Toba Batak ethnic communities before the entry of Christianity and Islam. They worship a God of justice and truth called *Debata Mulajadi Na Bolon*. In 1870, the hero Sisingamangaraja XII decided to direct the Parmalim belief because of the influence of the spread of Christianity and Islam in Batak Land. In the practice of worship (Figure-5), Parmalim believes in *Debata Mulajadi Na Bolon* as the creator of everything that exists, God Almighty.

Several Parmalim worship activities are routinely performed every year. During the ceremony, offerings are made of white Samosir goats and white and black chickens. The ceremony is accompanied by traditional dances and music of the Toba Batak tribe.

### Genetic distance analysis

The genetic distance between Samosir goats and other Indonesian local goats was analyzed based on the Cytb gene. Analyses were performed using the Kimura 2-parameter model [22]. There were a total of 1140 positions in the final dataset. The length of the Cytb sequences was 1140 bp, and the genetic distance between the populations ranged from 0% to 0.3% (Table-1). Several other types of local goats are very close to the Samosir goats (0.1%), including the Kacang, Jawarandu, Marica, and Gembrong goats.

**Table 1:** Estimates of evolutionary divergence over sequence pairs between groups.

	Samosir	Muara	Kacang	Jawarandu	Peranakan Etawah	Marica	Gembrong
Samosir							
Muara	0.002						
Kacang	0.001	0.001					
Jawarandu	0.001	0.003	0.002				
Peranakan Etawah	0.002	0.002	0.001	0.003			
Marica	0.001	0.001	0.000	0.002	0.001		
Gembrong	0.001	0.001	0.000	0.002	0.001	0.000	

### Phylogenetic relationship between the Samosir goat and other Indonesian local goat breeds

**Figure-6:**
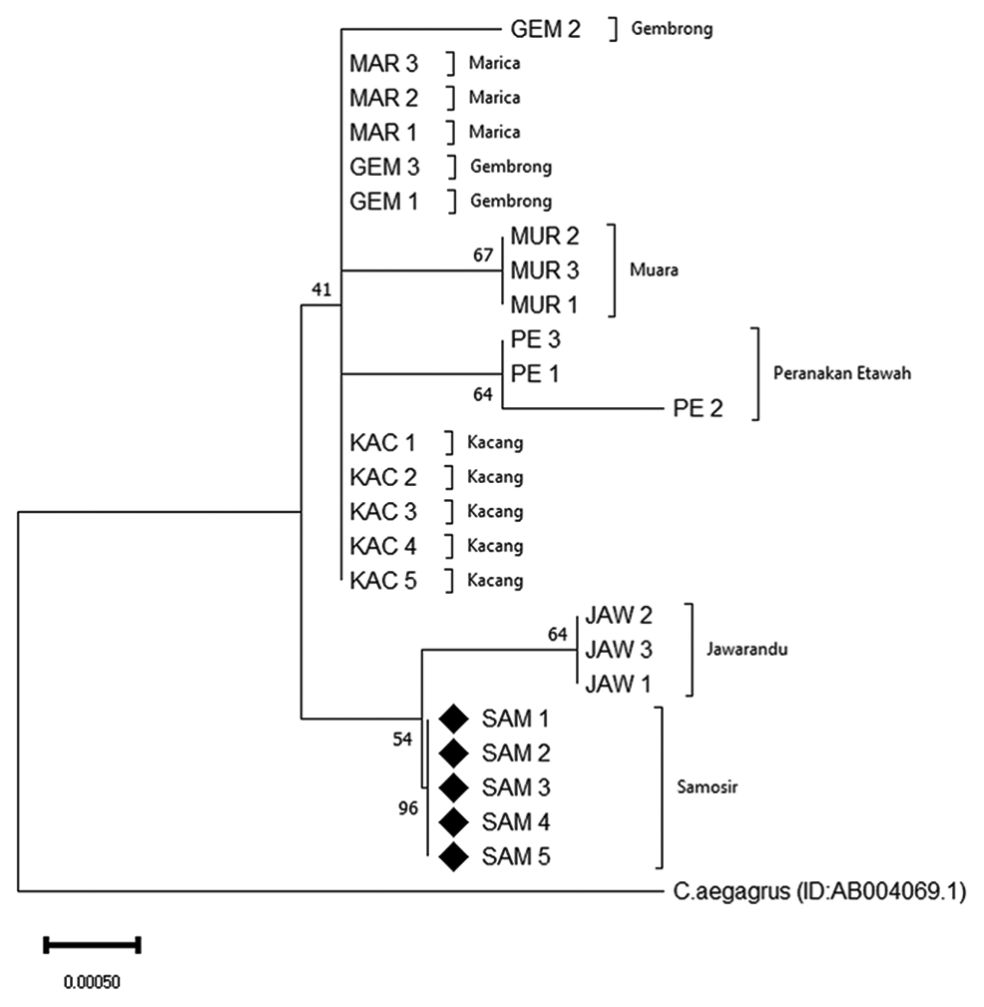
Phylogenetic tree of Samosir goat and other Indonesian local goat breeds.

The evolutionary history of the Sumatra goat was inferred with the use of the neighbor-joining method with 1000× repetition (bootstrap). The phylogenetic relationship showed that there were five clades. Samosir goats formed a clade and were separated from other goats (Figure-6). The result of this grouping was supported by high bootstrap values of 96% neighbor-joining. The Samosir goat had the closest genetic distance to the Jawarandu goat. Each individual of the Kacang, Gembrong, and Marica goats had a different haplotype.

## Discussion

### The uniqueness of the Samosir goat

The coat color of the Samosir goat population is predominantly white, with just a tiny fraction having a mixture of white with brown and black. This goat is supposed to be descended from the Kacang goat, which has long been adapted to the natural circumstances of Samosir and the selective intervention of the local people. The morphometric and genetic study revealed that Samosir goats have a relatively close genetic distance from Kacang goats [7,8,23]. Domesticated goats have been artificially selected to produce specialized elite breeds for milk, meat, and fibers, as well as breeds with distinct coat color phenotypes [24,25].

In ancient times, the Batak people thought that a pure white goat was a direct gift from God to its owner. According to the history and beliefs of the Batak people, white goats are used as an offering in religious ceremonies [26,27]. Because of the higher economic value and prestige of white goats, local people have historically preferred to selectively breed goats with uniform white coat color and keep them separate from other goats. One of the most extensively researched features of goats is coat color phenotype [28-30]. Changes in the phenotype of the Samosir goat in the population are a result of community selection for the desired characteristic [25,31,32]. A paucity of melanocytes in the skin and hair follicles causes white markings, white spots, and totally white phenotypes [32,33].

Samosir goats are unique to Samosir Island, where they were exclusively discovered and developed. The Kacang goat is the oldest indigenous goat in Indonesia and can be found almost throughout the Indonesian archipelago [7]. Phylogenetic analysis between Samosir goats and other local Indonesian goats showed that Samosir goats separated from other goats and formed a single clade. Geographic dispersion of a population and adaptation to its environment will result in the formation of a new form or subspecies [34,35]. Morphologically distinct, geographically separated groups are assumed to have speciated [36]. Phylogeographic studies based on mitochondrial DNA are increasingly used to evaluate sub-specific designations in various goat breeds [8,37,38].

### Breeding and conservation of the Samosir goat

Samosir goat breeding has historically been done conventionally and the population is shrinking. According to the latest census data collected by the Central Statistics Agency in 2015, the number of goats in Samosir Regency is 9700. Selection for a characteristic, whether direct or indirect, requires the existence of covariance between fitness and that trait, both in natural selection and in animal breeding [39]. Domestication and artificial selection have been linked to the fixation of causal genetic variations influencing breed-specific characteristics within areas of low genetic diversity, a phenomenon known as selection signatures or selective sweeps [40-42]. Samosir goats are subjected to undirected selection and continuous inbreeding in a small population. Inbreeding depression will occur from poor selection, which will have a negative impact on the productivity and reproduction of the goats [43-45].

The existence of a religious ceremony of the earliest Batak Toba tribe known as Parmalim signaled the beginning of the history of breeding Samosir goats. Goats are among the offerings used in Parmalim [26,46]. The goat must be completely white, as white goats have very high economic value and prestige. The Batak Toba tribe struggled to maintain traditional and cultural teachings before the introduction of new culture and religion by the Dutch through imperialism and colonialism [47]. Cultural developments and religious conversions in the Batak area have influenced the breeding of Samosir goats. The Parmalim belief is currently held by a small number of people. In 2020, 98.90% of the population of the Samosir Regency were Christian (Central Bureau of Statistics-Samosir Regency). At present, the primary reason for breeding Samosir goats is to provide meat and milk rather than religious ceremonial offerings. The white coat color is no longer the primary factor in selection and breeding.

## Conclusion

Phylogenetic analysis of Samosir goats and other indigenous Indonesian goats revealed that Samosir goats separated and formed a single clade, with a very close genetic distance from other local goats, such as the Kacang goat. The Toba Batak culture in Samosir Island has significantly influenced the selection and formation of the Samosir goat breed.

## Authors’ Contributions

SP and RW: Designed the study and collected goat samples for this study. SP and RW: Conducted the study in the laboratory. SP, RW, and WTA: Analyzed the data and wrote the manuscript. All authors have read and approved the final manuscript.
